# Context-Aware Evidence-Gated Plasticity for Multi-Goal Learning in Spiking Neural Networks

**DOI:** 10.21203/rs.3.rs-10157488/v1

**Published:** 2026-08-04

**Authors:** Samuel A Neymotin, Hananel Hazan, Gozde Unal, Christopher Earl, Haroon Anwar, Piotr J Franaszczuk, David Boothe

**Affiliations:** 1 Center for Biomedical Imaging and Neuromodulation, Nathan S. Kline Institute for Psychiatric Research, Orangeburg, NY; 2 Dept. Psychiatry, NYU Grossman School of Medicine, New York, NY; 3 Allen Discovery Center, Tufts University, Medford, MA; 4 Okinawa Institute of Science and Technology, Okinawa, Japan; 5 DEVCOM US Army Research Lab, Aberdeen, MD; 6 Dept. Neurology, Johns Hopkins University, Baltimore, MD

## Abstract

**Background / Introduction:**

Biologically inspired spiking neural networks can model adaptive behavior, but learning multiple goals is difficult because synaptic updates for different targets can interfere. We tested whether multi-timescale plasticity and context-specific credit assignment could improve continual multi-goal learning in a spiking navigation system inspired by entorhinal-hippocampal circuitry.

**Methods:**

We developed a closed-loop spiking model containing grid-like, place-like, target-related, association, and motor-output populations. An agent navigated in a two-dimensional environment with randomized starting locations and learned through reward-modulated spike-timing dependent plasticity (STDP/RL) and a novel evidence-gated plasticity (EGP) framework. EGP accumulates candidate synaptic modifications, evaluates them using reward evidence, and consolidates only changes that improve performance. A target-context variant maintained separate proposal stores and reward evaluation for each target.

**Results:**

STDP/RL learned and retained a single-target navigation policy, but multi-target training produced substantial interference, including attraction to incorrect targets after learning. Across 10 connectivity seeds, target-context EGP achieved higher late-stage reward than global EGP, improved weakest-target performance, and increased the fraction of targets achieving positive reward. In a longer continual-learning simulation, reward increased for all targets, TEST-phase performance increasingly exceeded TRAIN-phase performance, and proposal magnitudes grew over learning. Dwell-time confusion analyses showed that target-context EGP reduced wrong-target attraction and improved target selectivity relative to multi-target STDP/RL.

**Conclusions:**

These results demonstrate that spiking navigation circuits can learn goal-directed behavior using local plasticity, but robust multi-goal learning benefits from context-specific evidence-based consolidation. Target-context EGP provides a biologically motivated mechanism for reducing interference during continual reinforcement learning in spiking neural networks.

## Introduction

Animals navigate by combining spatial representations, sensory information, goal signals, and reinforcement feedback[1-4]. This ability depends on interactions among the entorhinal cortex, hippocampus, basal ganglia, and motor systems. Entorhinal grid cells provide a metric-like representation of space[5], while hippocampal place cells encode specific locations and support memory-guided behavior[6-8]. Together, these circuits enable animals to learn and execute routes to behaviorally relevant goals[9-11]. Understanding how such representations can drive adaptive behavior remains an important challenge for both computational neuroscience and biologically inspired artificial intelligence.

A large body of work has examined how grid-cell and place-cell representations emerge from recurrent dynamics, oscillatory mechanisms, attractor networks, sensory inputs, and learning[12-18]. Other studies have explored how hippocampal representations support reinforcement learning, planning, successor representations, and memory-guided decision-making[4]. Although these models have provided important insights into spatial coding and navigation, many focus primarily on representation rather than closed-loop behavior. In many cases, spatial codes are analyzed independently of an embodied agent, or learning is not required to continuously influence action selection, reward acquisition, and future synaptic change. Consequently, there remains a need for models that link biologically motivated spatial representations to adaptive behavior through mechanistic learning rules.

Spiking neural networks (SNNs) provide a useful framework for addressing this problem because they explicitly connect neural activity, synaptic plasticity, and behavior. Spike-timing dependent plasticity (STDP) offers a biologically plausible mechanism for modifying synapses according to the relative timing of pre- and postsynaptic activity[19-21]. When modulated by reward signals, STDP can support reinforcement learning in spiking circuits. Previous studies have shown that reward-modulated STDP can train spiking networks to perform sensorimotor tasks and learn goal-directed actions[22-26]. In spatial navigation models, STDP/RL can modify projections from spatial representations to motor circuits, allowing an agent to learn movements that approach a target.

In previous work, we developed spiking navigation models that coupled biologically inspired spatial representations to reinforcement-modulated plasticity[27-29]. These studies demonstrated that local learning rules can support goal-directed navigation, but also revealed a limitation: when multiple goals must be learned within a shared network, synaptic updates associated with one target can interfere with updates associated with another. Because overlapping spatial, association, and motor representations contribute to multiple navigation policies, online reward-modulated plasticity can produce competing weight changes that degrade previously learned behaviors. Such interference is related to continual-learning problems observed in artificial neural networks, but arises here within a biologically grounded spiking framework[30,31].

Biological learning systems appear to address similar challenges through plasticity mechanisms operating over multiple timescales[32,33]. Rapid STDP-like processes can encode recent activity patterns, whereas slower consolidation mechanisms determine which changes are retained[34]. Neuromodulatory signals, replay, synaptic tagging, sleep-dependent consolidation, and context-dependent gating have all been implicated in memory stabilization[35-38]. These observations suggest that robust multi-goal learning may require more than immediate online plasticity; it may require mechanisms that accumulate candidate synaptic changes, evaluate their behavioral consequences, and consolidate them in a context-dependent manner.

Here we extend our previous STDP/RL frameworks by introducing evidence-gated plasticity (EGP), a multi-timescale learning rule designed to reduce interference when multiple navigation goals must be learned within a shared network. Because different goals recruit overlapping spatial, association, and motor representations, learning one navigation policy can disrupt previously acquired behaviors. In EGP, candidate synaptic modifications are accumulated and subsequently consolidated only when reward evidence indicates that they improve performance. We further introduce a target-context EGP variant that maintains separate proposal stores and reward evaluation for different navigation goals before consolidation. Using a closed-loop spiking navigation model, we show that standard STDP/RL can learn and retain a single-target policy but exhibits substantial interference during multi-target learning. In contrast, target-context EGP improves multi-goal performance, increases target selectivity, and reduces wrong-target attraction by preserving context-specific credit assignment during consolidation.

## Methods

### Simulation environment and closed-loop navigation task

All simulations were implemented in Python using the NEURON simulation environment with NetPyNE for network specification, simulation control, and data output[39,40]. The spiking neural network was coupled to a two-dimensional navigation environment in which an agent moved through a bounded arena and attempted to reach one or more target locations. At each behavioral step, the environment provided the model with spatial and target-related inputs, the spiking network generated activity in motor-output populations, and the relative activity of those populations determined the agent’s next movement direction. The resulting state transition and reward were then used to update synaptic plasticity according to the learning rule used in that simulation.

The environment consisted of a two-dimensional square arena of size 100 x 100 pixels with 8 pixel padding to prevent edge artifacts. The agent’s position was represented by x- and y-coordinates. Targets were located at (24,24), (75,75), (24,75), (75,24), and (50,50). Single-target simulations used one fixed target, whereas multi-target simulations sequentially activated different targets across subepisodes. Each episode duration depended on simulation type and learning algorithm (see Training Protocols), and performance was quantified using target hits, cumulative reward, path trajectories, and dwell time near target locations.

At each simulation step, the agent selected one of four movement directions: north, south, east, or west. The selected action updated the agent’s position unless the move would cross the arena boundary or enter an invalid region, in which case the agent remained at or near its previous position and received an appropriate penalty. A target hit was recorded when the agent was positioned directly on the active target. Reward included positive reinforcement for target-directed movement and target interception, and negative reinforcement for movements away from the target (described further below).

### Event-based integrate-and-fire neuron model

Individual neurons were event-driven integrate-and-fire point-neuron models adapted from prior spiking network models used for sensorimotor reinforcement learning[24,25,41]. Each neuron had a membrane voltage state variable, a resting membrane potential, a spike threshold, an absolute refractory period, and additional state variables implementing after-hyperpolarization and relative refractoriness. When synaptic input events arrived, they changed postsynaptic voltage according to the synapse type and synaptic weight. If the membrane voltage crossed threshold and the neuron was not refractory or in depolarization blockade, the neuron emitted a spike and entered an absolute refractory period.

After each spike, an after-hyperpolarization variable was incremented and subtracted from the membrane voltage, then decayed exponentially back toward baseline. A relative refractory mechanism transiently increased spike threshold following a spike and decayed back to baseline with its own time constant. A depolarization block threshold prevented firing when the membrane voltage exceeded a high-voltage blockade value. This rule-based event-driven formulation allowed efficient simulation of large spiking networks while retaining several qualitative features of biological neurons, including refractoriness, spike-frequency adaptation, and depolarization block.

Each neuron also contained synaptic voltage variables corresponding to AMPA-like, NMDA-like, and GABAA-like synaptic inputs. Synaptic input events caused stepwise changes in these synaptic variables, which then contributed to the membrane voltage and decayed exponentially with synapse-specific time constants. AMPA-like inputs decayed rapidly, NMDA-like inputs decayed more slowly, and GABAA-like inputs provided inhibitory conductance-like effects. Synaptic delays were sampled from 1.8-2.2 and 2-4 ms depending on synapse type and projection class.

Neuron and synapse parameters were based on prior event-driven spiking models and adjusted to produce stable baseline firing rates in the present architecture. Excitatory, fast-spiking inhibitory, and low-threshold inhibitory neurons used separate parameter sets for resting membrane potential, spike threshold, depolarization block threshold, refractory time constants, after-hyperpolarization increments, and after-hyperpolarization decay constants. Parameter values are listed in Table 1.

### Spiking network architecture

**Figure 1.**
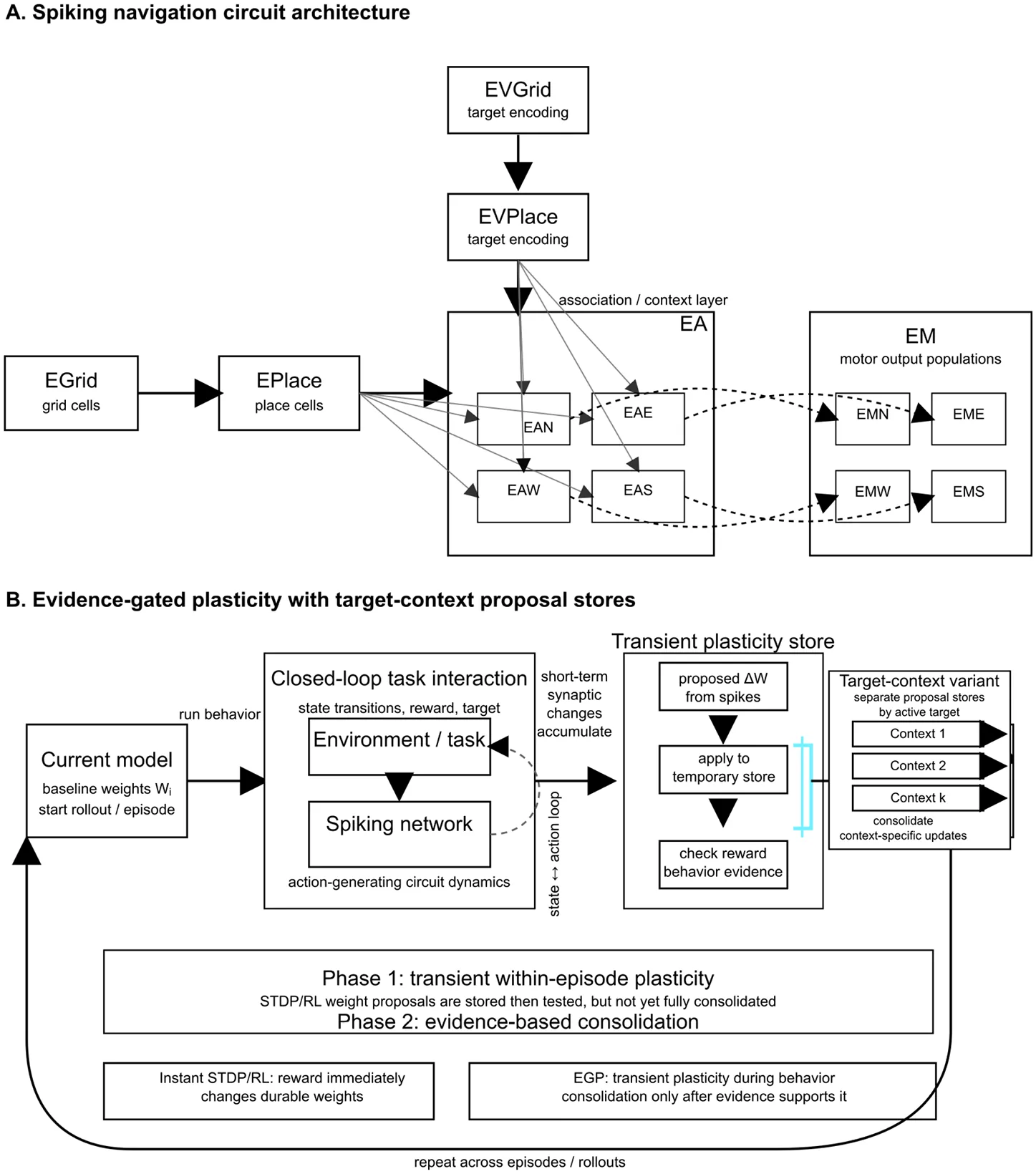
Spiking navigation circuit architecture and evidence-gated plasticity learning framework. A) **Schematic of the spiking neural network architecture used for closed-loop spatial navigation.** The agent’s current position is represented by grid-cell-like and place-cell-like populations (EGrid and EPlace). The currently active target is represented by target-encoding EVGrid and EVPlace populations. Spatial and target-context signals converge onto the excitatory association layer (EA), which is subdivided into action-pure subpopulations corresponding to north, east, west, and south movement directions. Each EA subpopulation projects to the matching excitatory motor-output population (EM): EAN→EMN, EAE→EME, EAW→EMW, and EAS→EMS. Activity in the EM populations determines the agent’s movement direction, allowing spatial and target-context representations to be transformed into direction-specific motor drive. **B) Schematic of evidence-gated plasticity (EGP).** During each rollout, the spiking network interacts with the environment in a closed loop: environmental state and target information drive network activity, motor-output activity selects actions, and the environment returns state transitions and reward. In standard online STDP/RL, reward-modulated spike-timing events immediately modify durable synaptic weights. In EGP, these spike- and reward-dependent weight changes are first accumulated as transient synaptic proposals. Proposed changes are evaluated using behavioral reward evidence and consolidated into durable weights only when supported by performance improvement. In the target-context variant, transient proposal stores are separated by active target context before consolidation, allowing context-specific updates to be evaluated and committed while reducing interference between navigation goals.

The model consisted of excitatory spatial representation populations, target/context populations, an association layer, motor-output populations, and inhibitory populations used to regulate network excitability (Fig. 1, Table 2). The overall architecture was designed to approximate a simplified entorhinal-hippocampal-to-motor transformation. Spatial inputs were represented by grid-like and place-like populations, target information was represented by target-encoding populations, and downstream association populations transformed spatial and target context into direction-specific motor drive.

The spatial input pathway included an excitatory grid-cell-like population, EGrid, and a downstream place-cell-like population, EPlace. EGrid encoded the agent’s current position using periodic two-dimensional spatial fields. For each EGrid neuron, a grid scale was sampled uniformly between 5 and 10 pixels. The grid spacing was then sampled as an integer between 5 and 7 times that scale, and the spatial phase offset was assigned by randomizing the starting x and y coordinates within the 100 × 100 environment. Grid fields were not restricted to a fixed set of prime scales or predefined modules. Instead, each neuron received an independently sampled periodic field. Grid fields were generated as hexagonally arranged Gaussian-like disks, with adjacent rows shifted by half the grid spacing and separated vertically by $\sqrt{3}∕2$ of the spacing.

Target-related (representing *visual*) input was represented by an analogous EVGrid population, which encoded the currently active target location using the same grid-map generation procedure. Thus, EGrid represented the agent’s current location, whereas EVGrid represented the current goal location in a comparable grid-like coordinate system. EGrid projected to EPlace, and EVGrid projected to EVPlace, producing downstream localized spatial and target-related representations. EPlace and EVPlace then projected to directional association populations, where current-location and targetlocation information could be integrated to guide motor-output activity.

The association layer consisted of excitatory association neurons (EA), subdivided into action-specific context populations: EAN, EAS, EAE, and EAW. These subpopulations were action-pure in the sense that each projected preferentially to the corresponding motor-output subpopulation. EAN projected to EMN, EAS to EMS, EAE to EME, and EAW to EMW. The motor layer consisted of excitatory motor-output populations encoding north, south, east, and west actions. At each behavioral step, spikes in the four EM populations were counted over the action-selection interval, and the action corresponding to the most active motor population was selected. If no population won or if multiple populations had equivalent activity, no move was made.

Inhibitory populations were included to prevent runaway excitation and to regulate firing rates in association and motor layers. Excitatory synapses were mediated by AMPA- and NMDA-like mechanisms, whereas inhibitory synapses were mediated by GABAA-like mechanisms. Plasticity was applied to EA→EM sub-populations. Unless otherwise stated, plasticity was limited to excitatory synapses onto excitatory postsynaptic cells.

### Background synaptic noise

Selected network populations received independent Poisson-distributed background synaptic input to maintain spontaneous activity and promote behavioral exploration. Noise was implemented using Poisson sources that generated AMPA-like excitatory input at 200 Hz and weaker GABA-like inhibitory input at 100 Hz. These inputs were delivered through fixed-weight synapses and were not subject to learning. Spatial and target-representation populations (EGrid, EVGrid, EPlace, and EVPlace), association populations (EA subpopulations), and most inhibitory populations were excluded from direct noise input. Consequently, background noise primarily influenced downstream action-selection and motor-output populations, providing variability in behavioral choices while leaving the spatial and target representations largely determined by external inputs and network interactions.

### Spatial and target input encoding

At each behavioral step, the agent’s current position determined the subset of active EGrid neurons. Active neurons received position-dependent stimulation with a peak firing rate of approximately 20 Hz. EPlace neurons received convergent input from EGrid and generated more localized place-like activity patterns. An analogous target-representation pathway, consisting of EVGrid and EVPlace populations, encoded the currently active goal location rather than the agent’s position. During multi-target training, the active target changed according to the training schedule, allowing EVGrid and EVPlace activity to provide downstream association populations with goal-specific context information. This target-context signal contributed to action selection and, in the target-context EGP model, was also used to separate accumulated synaptic proposals according to the currently active goal.

### Action selection

At each environment step, the network was simulated for 50 ms after spatial and target inputs were delivered. Spikes were counted in each motor-output population over this interval. Movement along the horizontal axis was determined by comparing activity in the eastward (EME) and westward (EMW) motor populations, whereas movement along the vertical axis was determined by comparing activity in the northward (EMN) and southward (EMS) populations. If one population in a pair produced more spikes than its opponent, the agent moved one step in the corresponding direction. If both populations in a pair produced identical spike counts, including zero spikes, no movement occurred along that axis. The selected action was sent to the environment, which updated the agent’s position and returned the next state and reward.

### Reward-modulated STDP

Standard learning simulations used reward-modulated spike-timing dependent plasticity (STDP/RL). This rule was adapted from prior work in which spike-pair timing generates an eligibility trace that is later converted into synaptic potentiation or depression by a scalar reward or punishment signal. When a postsynaptic neuron fired within a short temporal window (100 ms) after presynaptic activity, the corresponding synapse was tagged with a square pulse eligibility trace lasting 50 ms. This allows reinforcement delivered after the spike-pair event to modify synapses that have recently contributed to network activity.

For each eligible synapse, the reward-modulated weight change was computed as a product of the eligibility trace, a learning-rate parameter, and the critic/reward signal: $\Delta w_{ij}(t)=\eta_{STDP}\;*\;e_{ij}(t)\;*\;r(t)$ where $w_{ij}$ is the synaptic weight from presynaptic neuron i to postsynaptic neuron j, $e_{ij}(t)$ is the eligibility trace, r(t) is the critic or reward signal, and $\eta_{STDP}$ is the learning-rate parameter. Positive reward increased eligible synaptic weights, whereas negative reward decreased them. Weights were constrained to remain within values of 0-12. Plasticity was applied every 50 ms during online learning.

The critic signal was derived from the navigation environment. In single-target and multi-target STDP/RL simulations, the critic rewarded movements that improved navigation relative to the active target and penalized movements that increased distance from the target.

The reward signal depended only on whether the selected action moved the agent toward or away from the active target, with an additional bonus for target contact. The movement reward was defined as: $r_{move}(t)=+1$ if the action reduced distance to the target, $r_{move}(t)=-1$ if the action increased distance to the target, $r_{move}(t)=0$, if the distance to the target did not change.

Because reward scaling by distance was enabled, movement rewards were amplified near the target using a smooth distance-dependent gain: $S(d_{t})=1+e^{-{d_{t}}^{2}∕\lambda^{2}}$ with $d_{t}$ distance to the target, and λ=8. The critic signal applied to reward-modulated plasticity was therefore $R(t)=r_{move}(t)S(d_{t})+r_{hit}1_{hit}(t)$, where $r_{hit}=2$, and $1_{hit}(t)=1$ when the agent was on the active target and 0 otherwise.

Plasticity was restricted to action-specific association-to-motor projections: EAN→EMN, EAS→EMS, EAE→EME, and EAW→EMW. For each plastic synapse ($w_{ij}$), causal pre-before-post spike timing generated an eligibility trace, implemented as a fixed-duration square pulse. When a presynaptic spike preceded a postsynaptic spike within 100 ms, $0<{t_{j}}^{post}-{t_{i}}^{pre}\leq 100\;ms$ the synapse entered an eligible state for a duration of 50 ms. During this interval, subsequently arriving reward signals could modify the synaptic weight.

For eligible synapses, the reward-modulated weight change was $\Delta w_{ij}=A_{+}R(t)$ where R(t) is the critic signal and *A*_+_ = 0.045 is the reward-modulated STDP learning increment.

Eligibility tags were reset after reward delivery, every 50 ms, ensuring that reinforcement was assigned only to recent spike-pair events. In the target-specific motor learning rule, synapses contributing to the selected movement direction received the signed critic signal, whereas synapses associated with the opposing movement direction received the opposite sign scaled by 0.25. Candidate updates generated by this rule were either applied immediately in STDP/RL simulations or accumulated as proposals in evidence-gated plasticity simulations described below.

The rule was designed to enable the network to learn associations between spatial/target inputs, association-layer activity, and motor-output populations. In multi-target simulations, however, the same shared plastic synapses were modified across different target contexts, creating the possibility of interference between target-specific policies.

### Evidence-gated plasticity

Evidence-gated plasticity (EGP) was introduced to reduce the interference that occurred when STDP/RL updates from different targets were immediately committed to a shared synaptic matrix. In standard STDP/RL, eligible synapses are modified when a reward or punishment signal is delivered. EGP separates this process into two stages. First, local spike timing and reward-modulated eligibility generate candidate synaptic modifications during behavior. Second, these candidate modifications are evaluated using subsequent reward evidence and then consolidated into the durable synaptic matrix with a strength determined by that evidence.

For each plastic synapse, EGP maintained a transient proposal variable, $P_{ij}$, representing the accumulated candidate weight change since the previous consolidation event. During TRAIN phases, local STDP/RL events did not immediately modify the durable synaptic weight matrix. Instead, candidate changes were accumulated according to

$$P_{ij}\leftarrow P_{ij}+\Delta w_{ij}^{\text{proposal}},$$

where $\Delta w_{ij}^{\text{proposal}}$ was generated using the same reward-modulated spike-timing mechanism used by standard STDP/RL. Thus, EGP preserved the local credit-assignment structure of STDP/RL while delaying long-term consolidation.

After proposal accumulation, the model entered a TEST phase. During TRAIN phases, behavior was generated using the durable synaptic weight matrix W, while candidate synaptic modifications accumulated in a proposal store P without affecting ongoing action selection. In the target-context EGP variant, accumulated proposals were assigned to the currently active target-specific proposal store when target context changed, but they were not applied to the weights driving behavior during TRAIN. At the transition to TEST, the accumulated proposal state was temporarily added to the durable weights, allowing the network to evaluate behavior under the proposed synaptic state. TEST performance was then compared with the corresponding TRAIN performance to determine the degree of consolidation into the durable weight matrix.

Reward evidence was quantified using a normalized reward-improvement measure, $x=\frac{R_{TEST}-R_{TRAIN}}{|R_{TRAIN}|}$, where $R_{\text{TRAIN}}$ and $R_{\text{TEST}}$ denote the accumulated reward obtained during the TRAIN and TEST phases, respectively.

During TEST phases, the agent continued to navigate toward the same active targets used during TRAIN phases, and reward was accumulated from ongoing behavior in the environment. The TEST phase therefore differed from TRAIN primarily in how reward was used for proposal evaluation and consolidation rather than in the navigation task itself.

In the simulations reported here, reward accumulation additionally incorporated a need-based weighting mechanism. A running estimate of target-specific performance was maintained throughout learning, and targets exhibiting lower recent reward received larger need weights.

A running estimate of target-specific performance was maintained throughout learning using the average reward obtained while each target was active. Need weights were computed by comparing the recent performance of each target with the best- and worst-performing targets. Targets with lower recent reward received larger need weights, bounded between predefined minimum and maximum values, thereby increasing their influence on the TRAIN and TEST reward estimates. These weights scaled the contribution of reward samples to the TRAIN and TEST reward estimates, thereby increasing the influence of poorly performing targets on the evidence signal while retaining a single global proposal store.

Only positive reward improvements increased the evidence-dependent component of consolidation. EGP used the rectified sigmoid evidence function

g(x)={0,x<0,21+e−x−1,x≥0.}

This maps positive reward improvements onto the interval [0, 1), while preventing negative reward evidence from increasing the evidence-dependent component of consolidation.

The effective consolidation coefficient was then

$$\beta =\eta_{\text{EGP}}\;[\epsilon +(1-\epsilon )g(x)]\;,$$

where $\eta_{\text{EGP}}=1.0$ is the consolidation learning rate and *ϵ* = 0.05 is a minimum consolidation factor. The durable synaptic weight was updated according to

$$W_{ij}\leftarrow W_{ij}+\beta P_{ij}.$$

Consequently, all proposals received a small baseline level of consolidation, while proposals associated with larger improvements in TEST relative to TRAIN reward received progressively stronger consolidation. After consolidation, the proposal variables were reset and a new TRAIN-TEST cycle began.

This learning rule implements a functional separation between rapid local plasticity and slower evidence-dependent consolidation. Local STDP/RL generates candidate synaptic modifications on behavioral timescales, whereas EGP evaluates their utility using reward evidence before committing them to the durable synaptic matrix. In this manner, EGP approximates a distinction between rapid experience-dependent plasticity and slower consolidation processes that may occur during memory stabilization in biological systems. Conceptually, the EGP framework shares similarities with synaptic tagging and capture models, in which transient activity-dependent traces are stabilized only when supported by subsequent consolidation signals[42].

### Target-context evidence-gated plasticity

The target-context EGP variant was developed to prevent candidate synaptic modifications associated with different navigation goals from being mixed before consolidation. In global EGP, all accumulated proposals contribute to a single proposal variable (P_{ij}), regardless of which target is active. Consequently, updates that improve navigation toward one target can interfere with updates that improve navigation toward another target.

To address this limitation, target-context EGP maintains separate proposal stores for each target context, $P_{ij}^{\left(k\right)}$, where (k) indexes the currently active target. During TRAIN phases, reward-modulated STDP events generate candidate synaptic modifications exactly as in standard STDP/RL, but these updates are accumulated only within the proposal buffer associated with the active target: $P_{ij}^{\left(k\right)}\leftarrow P_{ij}^{\left(k\right)}+\Delta w_{ij}^{\text{proposal}}$.

When the active target changes during a TRAIN phase, the accumulated proposal generated under the previous target context is stored in the corresponding proposal buffer, and accumulation continues within the proposal buffer associated with the newly active target. Thus, candidate weight changes generated while pursuing different navigation goals remain separated throughout the TRAIN phase.

Each target context also maintains separate TRAIN and TEST reward estimates, $R_{\text{TRAIN}}^{\left(k\right)}$ and $R_{\text{TEST}}^{\left(k\right)}$. These were computed as the cumulative critic signal accrued while target (k) was active, normalized by the number of action intervals observed for that target. The context-specific reward-improvement measure was

$$x_{k}=\frac{{R^{\left(k\right)}}_{TEST}-{R^{\left(k\right)}}_{TRAIN}}{|{R^{\left(k\right)}}_{TRAIN}|}$$

When $R_{\text{TRAIN}}^{\left(k\right)}=0$, $x_{k}$ was set to 0 if TEST reward was also 0; otherwise, the denominator was replaced by $|R_{\text{TEST}}^{\left(k\right)}|$ to avoid division by zero.

As in global EGP, a rectified sigmoid transformed this reward-improvement measure into an evidence signal:

g(xk)={0,xk<0,21+e−xk−1,xk≥0.}

The target-context implementation also included a need-based weighting mechanism. For each target context, the model maintained a running estimate of recent target-specific performance. In the simulations reported here, this running performance estimate was updated from the raw environment reward obtained while the corresponding target was active. Contexts with lower recent performance received larger need weights, thereby increasing consolidation pressure for poorly performing targets. The bounded need weight for target *k*, denoted $n_{k}$, was computed from the relative performance of that target compared with the best and worst performing targets and constrained to remain within predefined limits.

The effective consolidation coefficient for target context *k* was therefore $\beta_{k}=n_{k}\eta_{\text{EGP}}\;[\epsilon +(1-\epsilon )g(x_{k})]$, where $\eta_{\text{EGP}}=1.0$ is the consolidation learning rate and *ϵ* = 0.05 is a minimum consolidation factor. Thus, $\beta_{k}$ is the effective evidence- and need-weighted consolidation coefficient applied to proposals generated under target context *k*.

The durable synaptic matrix was then updated using the weighted sum of all context-specific proposals:

$$W_{ij}\leftarrow W_{ij}+\sum_{k}\beta_{k}P_{ij}^{\left(k\right)}.$$

After consolidation, all context-specific proposal buffers were reset and a new TRAIN-TEST cycle began.

Importantly, target-context EGP preserves a single shared network architecture and a single durable synaptic weight matrix. Separation occurs only within the transient proposal stores and reward-evaluation process used during consolidation. This allows candidate modifications generated under different navigation goals to be evaluated independently before incorporation into long-term memory, thereby reducing interference between learned targets while preserving shared network representations.

## Training protocols

**Single-target STDP/RL** simulations were used to determine whether the network could learn a basic navigation policy. The agent was trained to reach a single central target at (50,50) for 800 episodes, with each episode lasting 187.5 seconds of simulated time. Performance was assessed using target hits, reward, and example trajectories. To test whether learning persisted after plasticity was disabled, the trained network was evaluated for 100 post-learning episodes with synaptic plasticity turned off. At the beginning of each episode, the agent was initialized to a random starting location in the 100 x 100 pixel environment, to allow the model to learn to reach the target from an arbitrary position.

**Multi-target STDP/RL** simulations used five fixed target locations: four quadrant targets at (24,24), (75,75), (24,75), and (75,24), and one central target at (50,50). Each episode used a single stationary target, and the active target was advanced sequentially across episodes. Starting location was randomized at the beginning of each episode. Each episode lasted 187.5 s, and the full learning phase contained 4000 episodes, corresponding to 800 episodes per target. The active target was represented by EVGrid and EVPlace input. STDP/RL remained active during the learning phase. After learning, synaptic plasticity was disabled and the network was evaluated to determine whether target-dependent navigation policies had been retained in the learned synaptic weights.

**Short multi-seed EGP** simulations compared global EGP with target-context EGP across 10 independently seeded network instances. The global EGP batch used a single proposal store, whereas the target-context EGP batch used target-context EGP, in which proposal buffers and reward evidence were separated by target context. Both EGP modes used need-based reward weighting, with need weights computed from recent target-specific performance and bounded between 0.75 and 2.0. In the target-context implementation, these need weights additionally scaled the context-specific consolidation coefficients. These simulations used five target locations, (24,24), (75,75), (24,75), (75,24), and (50,50). Targets and randomized starting locations were updated every 150 action intervals, corresponding to 7.5 s of simulation time, because each action interval lasted 50 ms. Each TRAIN or TEST phase lasted 750 action intervals, or 37.5 s, and therefore contained five 7.5 s target blocks. Each 375 s episode contained repeated alternating TRAIN and TEST phases, and 800 episodes were run for each seed. Across seeds, the connectivity seed was varied from 1 to 10, while the EGrid (grid-cell) and EVGrid (target encoding grid cells) seeds were varied in parallel.

**To characterize the long-timescale behavior of target-context EGP**, an extended simulation was performed using a single network realization (connectivity seed 1, grid-cell seed 9876, EV-cell seed 9876). The simulation consisted of 2000 sequential learning episodes. Prior to each episode, a new starting location was selected randomly from valid locations within the environment. Five target locations were used throughout learning: (24,24), (75,75), (24,75), (75,24), and (50,50). The target-context EGP implementation maintained separate proposal stores, reward estimates, and consolidation coefficients for each target context, and incorporated need-based weighting to increase consolidation pressure for underperforming targets.

Each episode lasted 1500 s and consisted of alternating TRAIN and TEST phases. TRAIN and TEST phases each lasted 150 s (3000 action intervals of 50 ms). Within both phases, the active target context and associated starting location changed every 30 s (600 action intervals), resulting in five target blocks per TRAIN phase and five target blocks per TEST phase. Consequently, each episode contained five TRAIN phases and five TEST phases, yielding 25 TRAIN target blocks and 25 TEST target blocks per episode. Candidate synaptic modifications accumulated separately within each target context during TRAIN phases, while context-specific reward evidence obtained during TEST phases determined the degree of consolidation into the durable synaptic weight matrix. These simulations were used to examine long-timescale reward dynamics, context-specific consolidation, trajectory evolution, and the emergence of stable target-selective navigation behavior.

### Behavioral and statistical analyses

Performance was quantified using several complementary metrics. Target hits measured the number of successful target interceptions per episode or subepisode. Reward measured the cumulative critic signal over TRAIN or TEST phases. Learning curves were smoothed using moving averages over 100 episodes (unless stated otherwise). Multi-seed EGP comparisons were summarized as mean ± SEM across connectivity seeds. Because the same connectivity seeds were evaluated under both global and target-context EGP conditions, paired Wilcoxon signed-rank tests were used to assess differences in late-stage reward, weakest-target reward, and the fraction of targets achieving positive reward. Statistical significance was defined as p < 0.05.

Target-specific performance was assessed by computing late-stage reward for each target over the final 100 episodes. Weakest-target reward was defined as the minimum target-specific reward for a given seed. The fraction of targets with positive late-stage reward was used to quantify how many targets were successfully learned.

To quantify target-target interference, we computed dwell-time confusion matrices. For each active target, we measured the fraction of time the agent spent within radius r (5 pixels) of each possible target location. Matrix rows corresponded to the active target, and columns corresponded to the target location near which the agent was located. Diagonal entries therefore measured dwell time near the correct target, whereas off-diagonal entries measured attraction to incorrect targets. Summary metrics included mean diagonal dwell, weakest-target diagonal dwell, maximum off-diagonal dwell, and the ratio of mean diagonal to mean off-diagonal dwell.

Trajectory plots were generated to visualize behavior during early learning, late learning, and post-learning evaluation. For each trajectory, the active target was indicated separately from inactive targets, and the agent’s position over time was color coded to show movement through the episode.

### Software and Computational Resources

The model was implemented in the NetPyNE modeling platform and run using the NEURON simulation environment [39,40]. Simulations were performed on multiple high performance compute servers. A server equipped with Intel Xeon Platinum 2.9 GHz CPUs using 30 cores and 503 GB of RAM took 59 s to run a 187.5 s simulation with STDP/RL turned on. Total compute time across all simulation types presented in the manuscript is shown in Table 4. Source code, simulation scripts, and visualization tools will be made publicly available through GitHub and ModelDB [43] after publication.

## Results

### A spiking navigation circuit learns a single target using reward-modulated STDP

**Figure 2.**
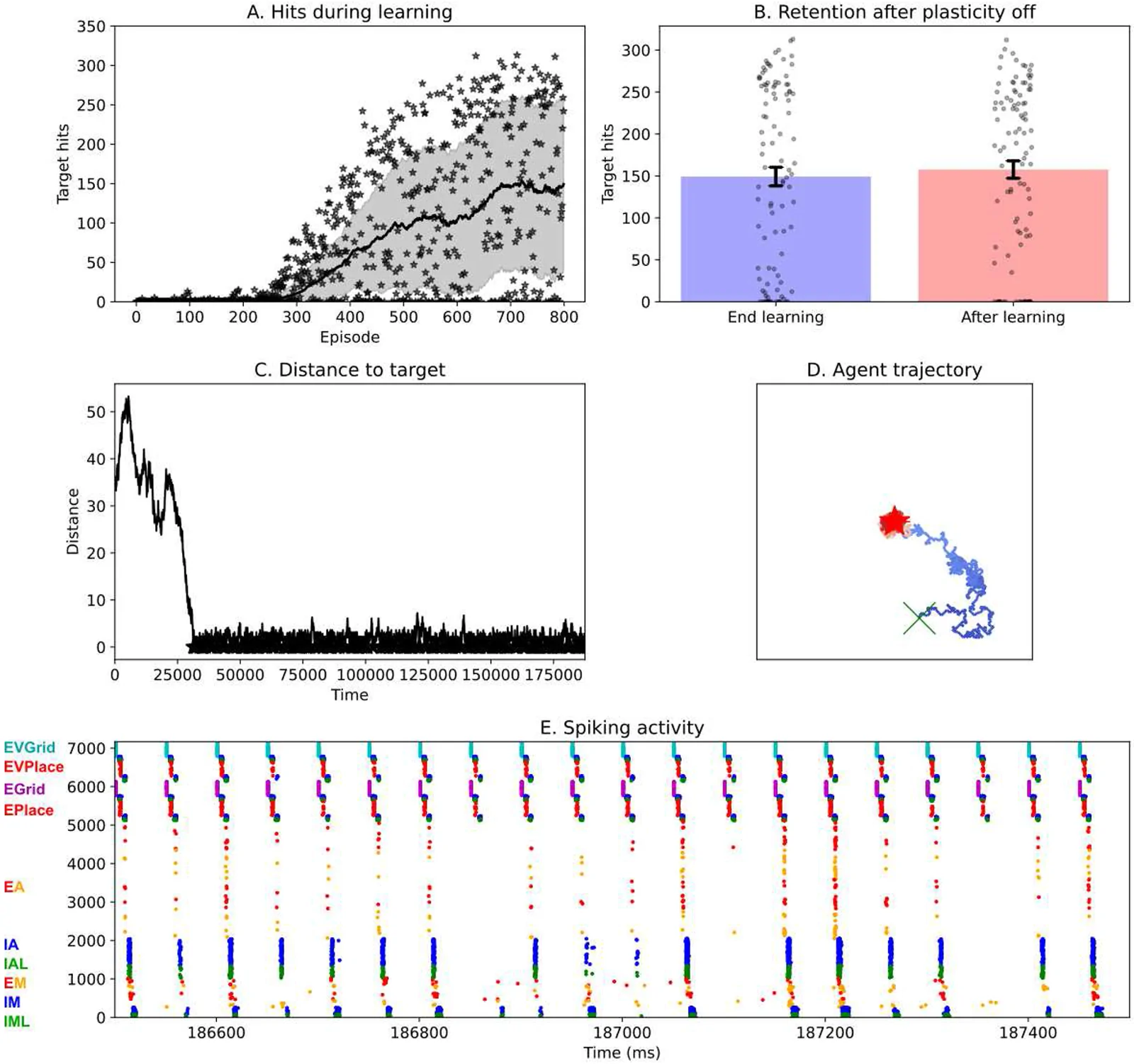
Single-target navigation learning and retention using reward-modulated STDP. **A)** Number of target hits as a function of training episode during learning of a single navigation target. Individual points represent target hits obtained during each episode. The solid black line shows the moving average (n=100 episodes) across episodes, and the shaded region indicates ± standard deviation (n=100). Target hits increase substantially during training, demonstrating acquisition of a goal-directed navigation policy. **B)** Comparison of target hits during the final 100 learning episodes (“End learning”) and the first 100 episodes after plasticity was disabled (“After learning”). Individual points represent episodes, bars show mean performance, and error bars indicate standard error of the mean. Similar performance before and after plasticity was disabled indicates that the learned navigation policy was retained in the synaptic weights. **C)** Distance between the agent and the target during a representative post-learning episode. The distance decreases rapidly as the agent approaches the target and remains near zero following repeated target interceptions. **D)** Representative post-learning trajectory of the agent in the two-dimensional environment. The green X indicates the starting location and the red star indicates the target location. The trajectory is color coded from blue (early) to red (late) to illustrate progression through the episode. **E)** Raster plot of spiking activity during the representative post-learning episode. Spatial representation populations (EGrid, EPlace), target-context populations (EVGrid, EVPlace), association neurons (EA), motor-output populations (EM), and inhibitory populations exhibit coordinated activity that transforms spatial and target information into goal-directed motor behavior.

We first tested whether the model could learn a simple goal-directed navigation policy using standard reward-modulated spike-timing dependent plasticity (STDP/RL). In these simulations, the agent navigated in a two-dimensional environment containing a single target, while plasticity modified synaptic projections from spatial representation populations to downstream motor-output circuits. Across training episodes, the number of target hits increased substantially, indicating that the network learned to transform its spatial and target-related activity into actions that brought the agent toward the goal (Fig. 2A).

To determine whether learning reflected durable changes in synaptic weights rather than only transient online adaptation, we next evaluated performance after learning was disabled. The agent maintained similar target-reaching performance when plasticity was turned off, indicating that the learned policy was stored in the synaptic configuration of the network rather than requiring ongoing weight updates during each episode (Fig. 2B). Consistent with this interpretation, plastic synaptic weights increased over training 4-5x on average (Fig. S1). Example post-learning episodes showed trajectories directed from the starting location toward the target, with repeated target interceptions visible in the distance-to-target trace (Fig. 2C,D). Raster plots revealed coordinated activity across spatial representation populations (EGrid, EPlace), target-related populations (EVGrid, EVPlace), association neurons (EA), and motor-output populations (EM), demonstrating how spatial and target information propagated through the circuit to generate goal-directed actions (Fig. 2E).

Together, these results demonstrate that the spatial navigation architecture and reward-modulated STDP learning rule are sufficient to acquire and retain a single-target navigation policy. The persistence of performance after plasticity was disabled indicates that learning produced stable synaptic changes capable of supporting behavior without continued online adaptation.

### Multi-target learning with STDP/RL produces interference between learned target policies

**Fig. 3.**
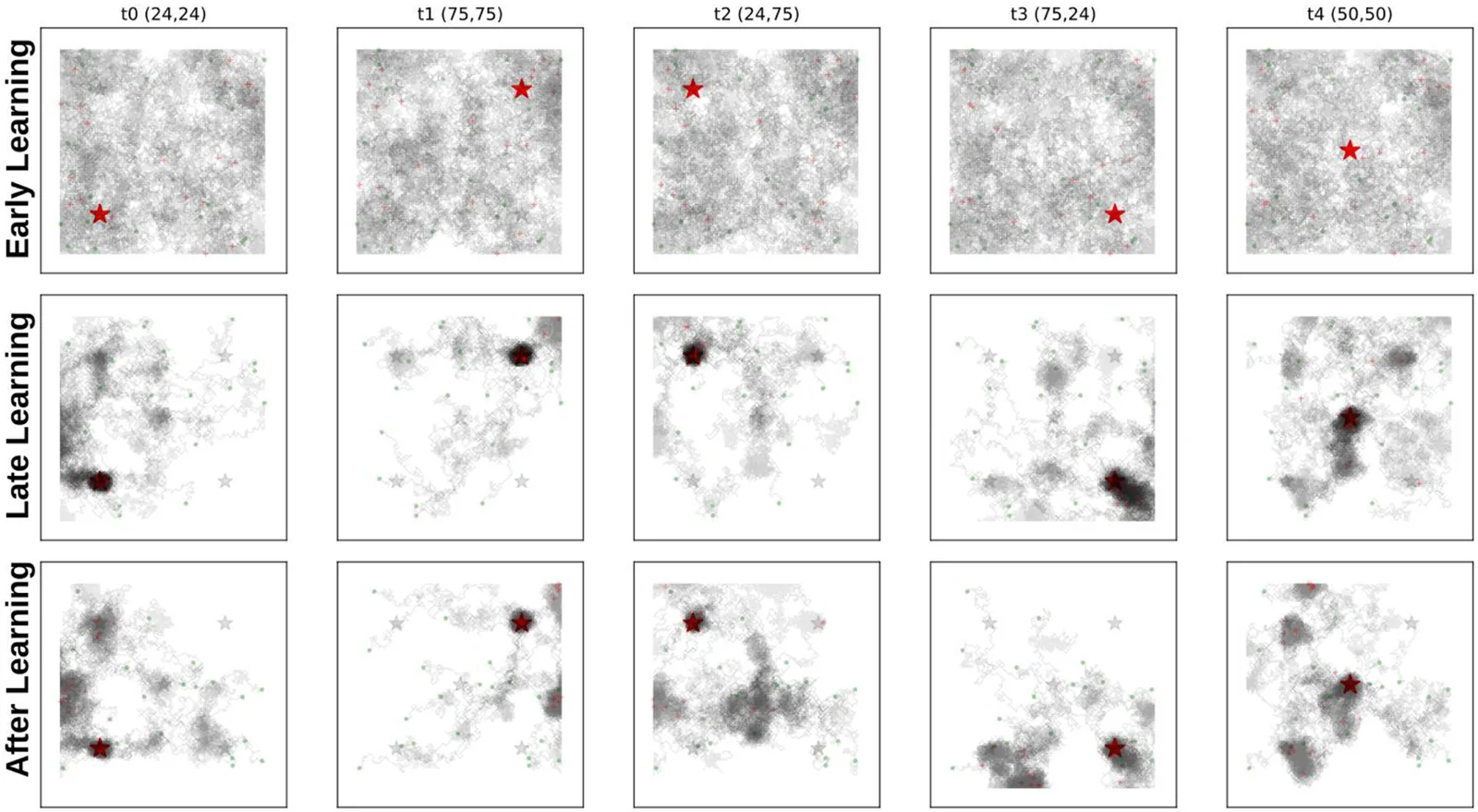
Agent trajectories during multi-target STDP/RL learning. Representative trajectories from a multi-target STDP/RL simulation shown for five target locations (columns) at three stages of learning (rows). **Early Learning** shows exploratory behavior with little target selectivity. **Late Learning** shows increased occupancy near the active target, indicating successful acquisition of target-directed navigation policies while STDP/RL remains active. However, trajectories continue to exhibit attraction toward previously learned targets, consistent with emerging target-target interference. **After Learning** shows performance following removal of online plasticity. Although the agent generally retains the ability to navigate toward the active target, interference becomes more pronounced, with trajectories more frequently deviating toward competing target locations. Red stars indicate the active target, gray stars indicate inactive targets, green circles indicate episode starting locations, and red “X” symbols indicate final agent positions.

We next asked whether the same online STDP/RL mechanism could support learning across multiple target locations. In these simulations, the agent was trained on five targets distributed across the environment. Early in training, trajectories were broadly exploratory and did not show consistent attraction to the active target. By late learning, trajectories became more directed toward the current target, indicating that the network acquired target-dependent navigation policies. However, the learned behavior remained unstable across targets and became especially vulnerable when plasticity was turned off (Fig. 3).

**Fig. 4.**
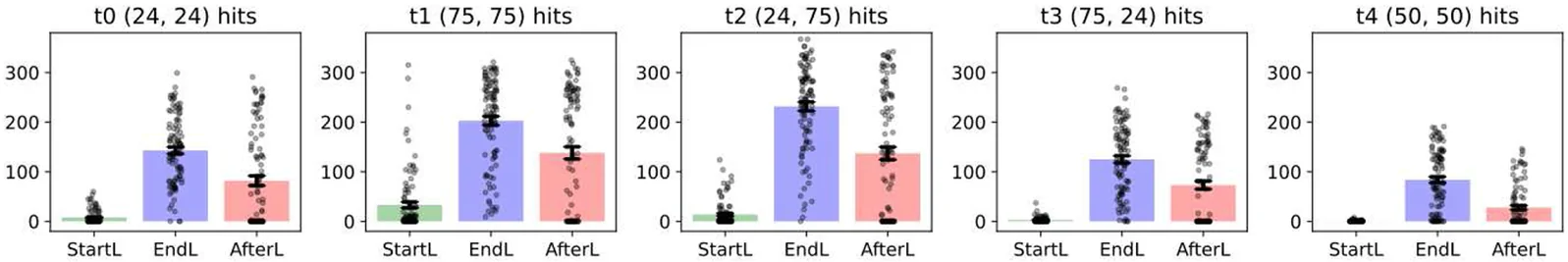
Multi-target STDP/RL learning produces target-dependent performance and interference. Target hits for each of the five navigation targets during **Early Learning** (first 100 episodes: “*StartL*”), **Late Learning** (last 100 learning episodes: “*EndL*”), and **After Learning** (100 evaluation episodes following removal of plasticity: “*AfterL*”). Performance improved substantially during learning for all targets, demonstrating that STDP/RL can acquire multiple target-directed navigation policies. However, performance varied substantially across targets and declined during the post-learning evaluation phase, consistent with target-target interference arising from storage of multiple navigation policies within a shared synaptic substrate. Individual points represent the number of target hits per episode, bars indicate the mean, and error bars indicate the standard error of the mean.

Quantitative analysis of target hits confirmed this pattern (Fig. 4). During learning, the number of target hits increased for all five targets, but performance differed substantially across target locations. After learning was disabled, target-reaching performance declined for several targets, indicating that online plasticity had been partially compensating for interference between target policies during training. Thus, although STDP/RL could support learning of multiple targets, the learned policies were not cleanly separated and remained susceptible to target-target interference.

**Fig. 5.**
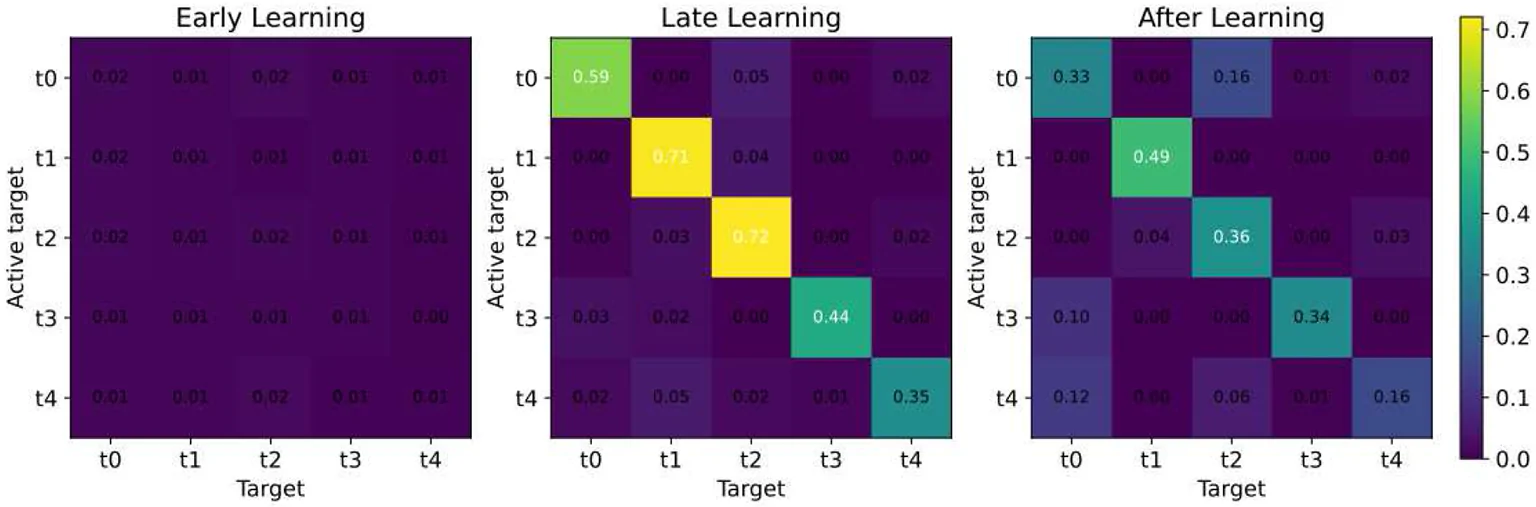
Dwell-time confusion matrices reveal target-target interference during and after multi-target STDP/RL learning. Confusion matrices show the fraction of each episode spent within a 5-pixel radius of each target location. Rows correspond to the active target (the current goal represented by EV and EVPlace populations), and columns correspond to the target location occupied by the agent. Diagonal entries therefore indicate correct target-specific occupancy, whereas off-diagonal entries indicate attraction to incorrect targets. **Early Learning** (left) shows little target selectivity and broadly distributed occupancy across targets. **Late Learning** (center) exhibits increased diagonal structure, indicating successful acquisition of target-directed navigation behavior. **After Learning** (right) shows reduced diagonal dominance and increased off-diagonal occupancy, demonstrating greater target confusion following reduction or removal of online plasticity. These results indicate that although STDP/RL can acquire multiple navigation policies, interference between target representations remains substantial and contributes to performance degradation after learning.

To further quantify this interference, we measured the fraction of each episode that the agent spent near each target location (Fig. 5). Under ideal target-specific behavior, dwell time should be concentrated along the diagonal of the matrix, corresponding to time spent near the active target. Instead, multi-target STDP/RL produced off-diagonal dwell, especially after learning was turned off, indicating attraction to incorrect target locations. These results show that standard online STDP/RL can learn useful target-directed behavior, but does not reliably prevent interference between multiple learned navigation goals.

### Evidence-gated plasticity improves multi-target learning

The multi-target STDP/RL results suggested that immediate online plasticity was insufficient to robustly separate credit assignment across target contexts. We therefore introduced evidence-gated plasticity (EGP), a two-phase learning rule in which candidate synaptic modifications accumulate during TRAIN phases and are consolidated only when subsequent TEST performance provides evidence that the proposed changes improve behavior (see Methods). In both EGP variants, need-based reward weighting increased the influence of underperforming targets during reward evaluation. This framework was designed to reduce noisy or destabilizing updates by evaluating accumulated plasticity over longer timescales while encouraging learning of poorly performing targets.

**Fig. 6.**
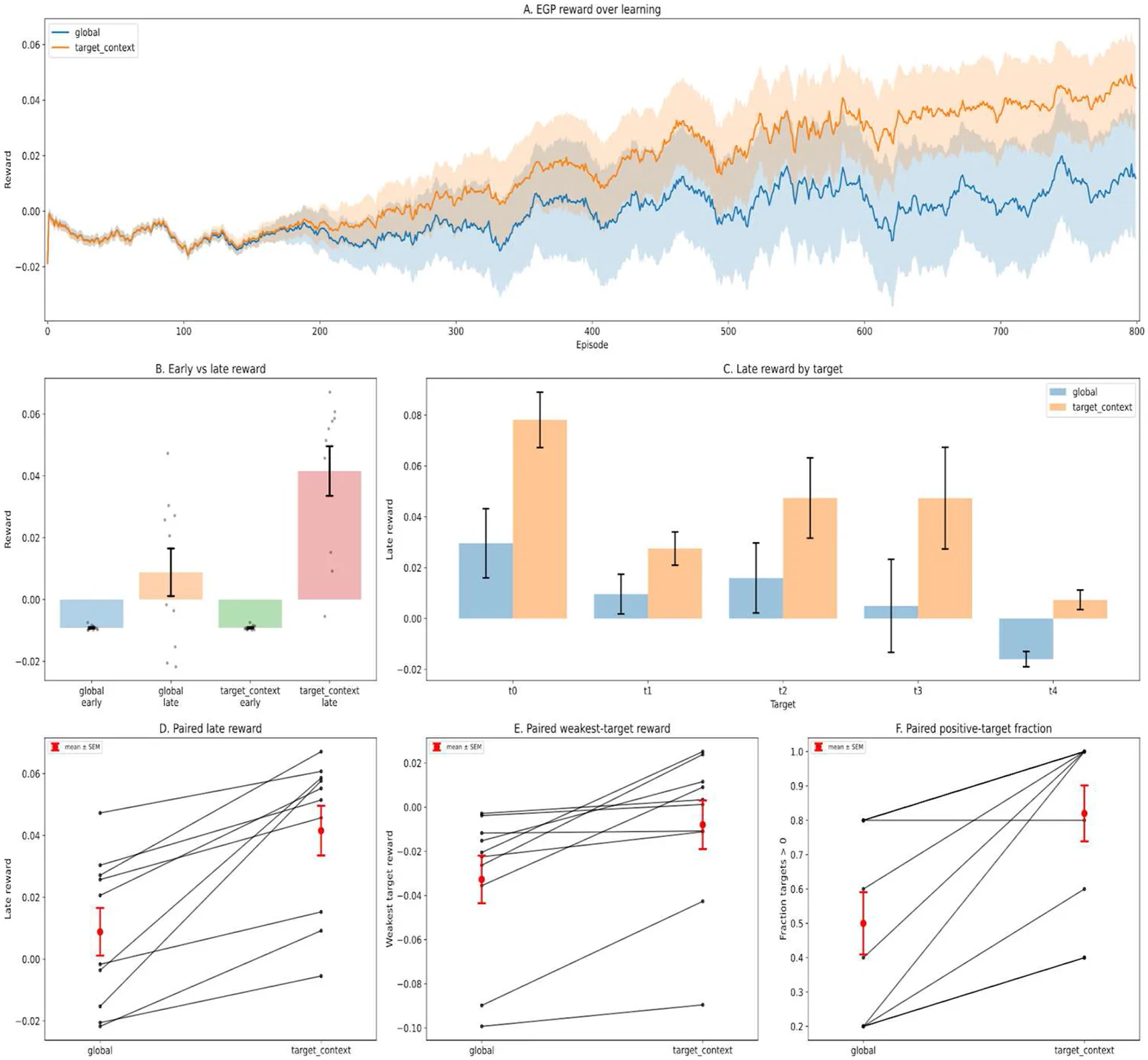
Target-context evidence-gated plasticity (EGP) improves multi-target learning relative to global EGP. Comparison of global EGP and target-context EGP across 10 independently seeded network realizations. Both methods employed evidence-gated consolidation and need-based reward weighting, but target-context EGP maintained separate proposal stores, reward estimates, and consolidation signals for each target context. **A)** Mean TEST-phase reward across learning episodes. Shaded regions indicate ± SEM across connectivity seeds. Both methods improved reward over training, while target-context EGP achieved substantially higher reward during later stages of learning. **B)** Early (first 100 episodes) and late (last 100 episodes) TEST-phase reward distributions across seeds. Individual points represent connectivity seeds; bars indicate mean ± SEM. Initial performance was similar across conditions, whereas target-context EGP achieved substantially higher late-stage reward. **C)** Mean late-stage reward for each target location, averaged across the final 100 episodes and all seeds. Target-context EGP improved reward for all five navigation targets relative to global EGP, indicating more effective learning across multiple goals. **D)** Paired comparison of late-stage reward for matched connectivity seeds. Each line connects the performance of a single seed under global and target-context EGP. Target-context EGP improved late-stage reward in all 10 connectivity seeds (Wilcoxon signed-rank test, p = 0.00195). **E)** Paired comparison of weakest-target reward for each seed. Target-context EGP improved weakest-target reward in all 10 connectivity seeds (Wilcoxon signed-rank test, p = 0.00195), indicating reduced target-specific failures and more balanced learning across goals. **F)** Paired comparison of the fraction of targets achieving positive late-stage reward. Target-context EGP increased the fraction of targets achieving positive reward in 9 of 10 connectivity seeds (Wilcoxon signed-rank test, p = 0.00742). Together, these results demonstrate that separating proposal accumulation, reward evaluation, and consolidation by target context improves multi-target learning beyond a globally mixed EGP rule. Improvements were observed not only in average reward, but also in weakest-target performance and the number of successfully learned targets, indicating reduced interference and more robust acquisition of multiple navigation policies.

We first compared global EGP, in which accumulated plasticity was pooled across target contexts, with target-context EGP, in which candidate synaptic changes, reward estimates, and consolidation signals were maintained separately for each target context before consolidation. Both methods began with nearly identical early reward, indicating that task difficulty and initial performance were closely matched across conditions. Over learning, however, target-context EGP produced substantially higher late-stage reward than global EGP (Fig. 6A,B). This improvement was consistent across matched connectivity seeds, with most seeds showing higher late-stage reward under target-context EGP than under global EGP (Fig. 6D).

Target-context EGP also improved target-specific learning. Late-stage reward was higher for every target under target-context EGP than under global EGP (Fig. 6C). The weakest-target reward improved across seeds, indicating that target-context EGP reduced the severity of the most difficult target-specific failures (Fig. 6E). In addition, the fraction of targets achieving positive late-stage reward increased under target-context EGP (Fig. 6F). Both EGP variants employed need-based reward weighting, so the improvement under target-context EGP is unlikely to reflect preferential reinforcement of poorly performing targets alone. We note, however, that need weighting enters the two variants at different points: scaling the reward estimates that feed a single proposal store in global EGP, versus scaling the per-context consolidation coefficients in target-context EGP, so the conditions are matched in the presence of need weighting but not in its precise mechanism. With this caveat, the results indicate that maintaining separate proposal stores and reward-evaluation processes for different target contexts improves multi-target learning beyond a globally mixed EGP rule.. Thus, separating candidate plasticity and credit assignment by target context reduced interference between navigation goals while preserving the benefits of evidence-based consolidation.

Paired Wilcoxon signed-rank tests confirmed these improvements across matched connectivity seeds. Target-context EGP increased mean late-stage reward from 0.0088 ± 0.0077 SEM to 0.0416 ± 0.0080 SEM (W = 0, p = 0.00195), improved weakest-target reward from −0.0328 ± 0.0108 SEM to −0.0080 ± 0.0110 SEM (W = 0, p = 0.00195), and increased the fraction of targets achieving positive reward from 0.50 ± 0.09 SEM to 0.82 ± 0.08 SEM (W = 0, p = 0.00742). Target-context EGP improved late-stage reward and weakest-target reward in all 10 connectivity seeds and improved the fraction of successful targets in 9 of 10 seeds.

### Target-context EGP supports learning across all targets but leaves residual target-dependent variability

**Fig. 7.**
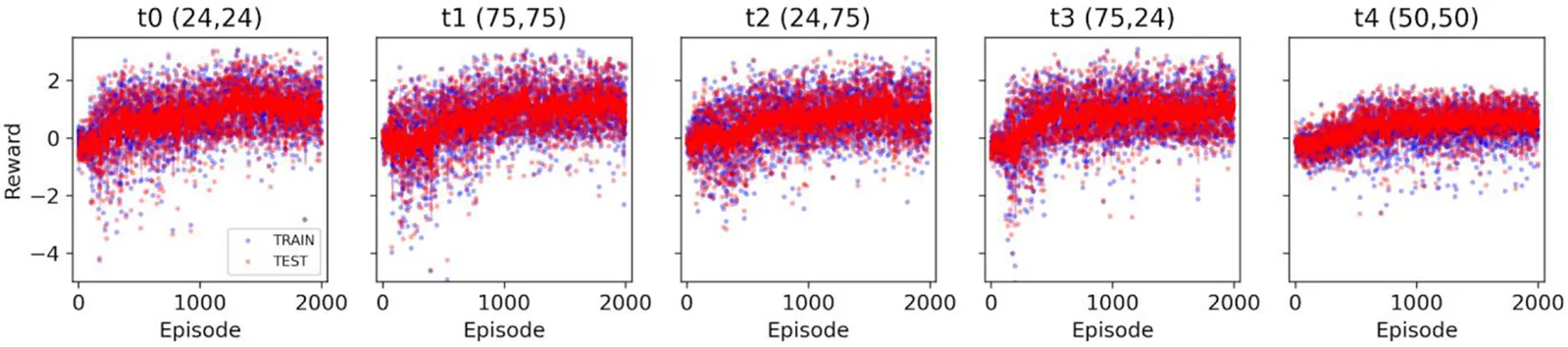
Target-specific reward dynamics during target-context evidence-gated plasticity (EGP). Reward is shown separately for each of the five navigation targets over the course of learning. Blue points indicate TRAIN-phase reward and red points indicate TEST-phase reward. Solid lines show moving averages computed across episodes. All targets exhibit progressive reward improvement over training, demonstrating successful acquisition of target-directed navigation policies under target-context EGP. As learning proceeds, TEST-phase reward often matches or exceeds TRAIN-phase reward, indicating that accumulated synaptic proposals are successfully consolidated into durable navigation policies rather than producing only transient within-phase improvements. Although reward increases for all targets, differences remain across target locations, reflecting residual variability in learning difficulty despite context-specific credit assignment. The central target (t4; 50,50) achieves lower asymptotic reward than the quadrant targets, suggesting that target-context EGP substantially reduces, but does not completely eliminate, interference and competition among navigation goals.

To examine learning dynamics in more detail, we analyzed a longer target-context EGP simulation. Reward increased over training for all five target locations, in both TRAIN and TEST phases (Fig. 7). This indicates that target-context EGP supported acquisition of goal-directed navigation policies across the full target set. However, performance was not completely uniform across targets. The central target showed lower asymptotic reward than the quadrant targets, suggesting that context-specific credit assignment mitigated but did not fully eliminate target-dependent differences in learning and navigation efficiency.

**Fig. 8.**
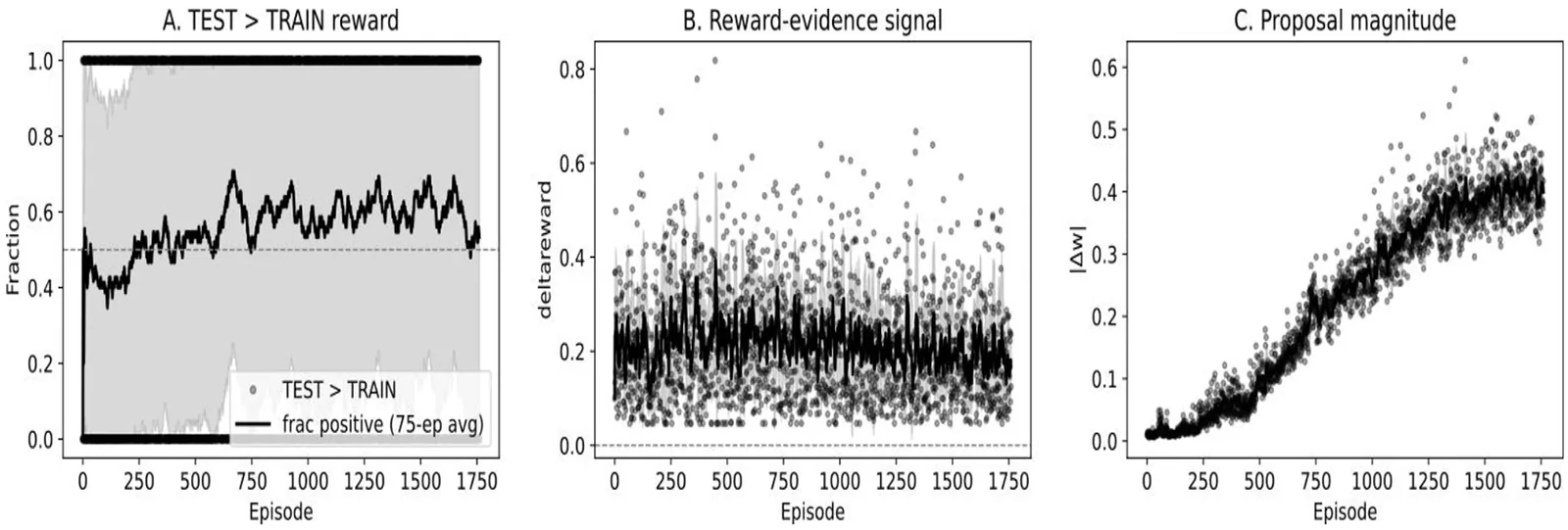
Mechanisms underlying target-context evidence-gated plasticity (EGP). **A)** Fraction of target-context evaluations in which TEST-phase reward exceeded TRAIN-phase reward. The moving average rises above chance levels (0.5, dashed line), indicating that accumulated synaptic proposals increasingly produce performance improvements when evaluated during TEST phases. **B)** Reward-evidence signal (ΔR), representing the normalized difference between TEST and TRAIN reward that determines the strength of proposal consolidation. Positive values indicate that accumulated proposals improved performance and therefore receive stronger consolidation. **C)** Mean magnitude of accumulated synaptic proposals (∣ΔW) across contexts. Proposal magnitudes increase over learning as candidate synaptic modifications accumulate within target-specific proposal stores. Together, these measures illustrate the three stages of the EGP algorithm: generation of candidate synaptic modifications during TRAIN phases, evaluation of their behavioral consequences during TEST phases, and evidence-dependent consolidation of beneficial proposals into the durable synaptic weight matrix.

We next examined the internal signals used by EGP to regulate consolidation. The fraction of episodes in which TEST reward exceeded TRAIN reward rose above chance over learning, indicating that accumulated candidate updates increasingly improved performance when evaluated in the test phase (Fig. 8A). The reward-evidence signal remained positive across training, showing that proposed synaptic modifications generally produced beneficial changes in behavior (Fig. 8B). The magnitude of accumulated weight proposals also increased during learning, consistent with the development of increasingly structured synaptic modifications as the network acquired target-dependent navigation policies (Fig. 8C). These results illustrate the core operation of EGP: candidate synaptic changes accumulate, their behavioral consequences are evaluated, and beneficial changes are selectively consolidated.

### Target-context EGP reduces wrong-target attraction

**Fig. 9.**
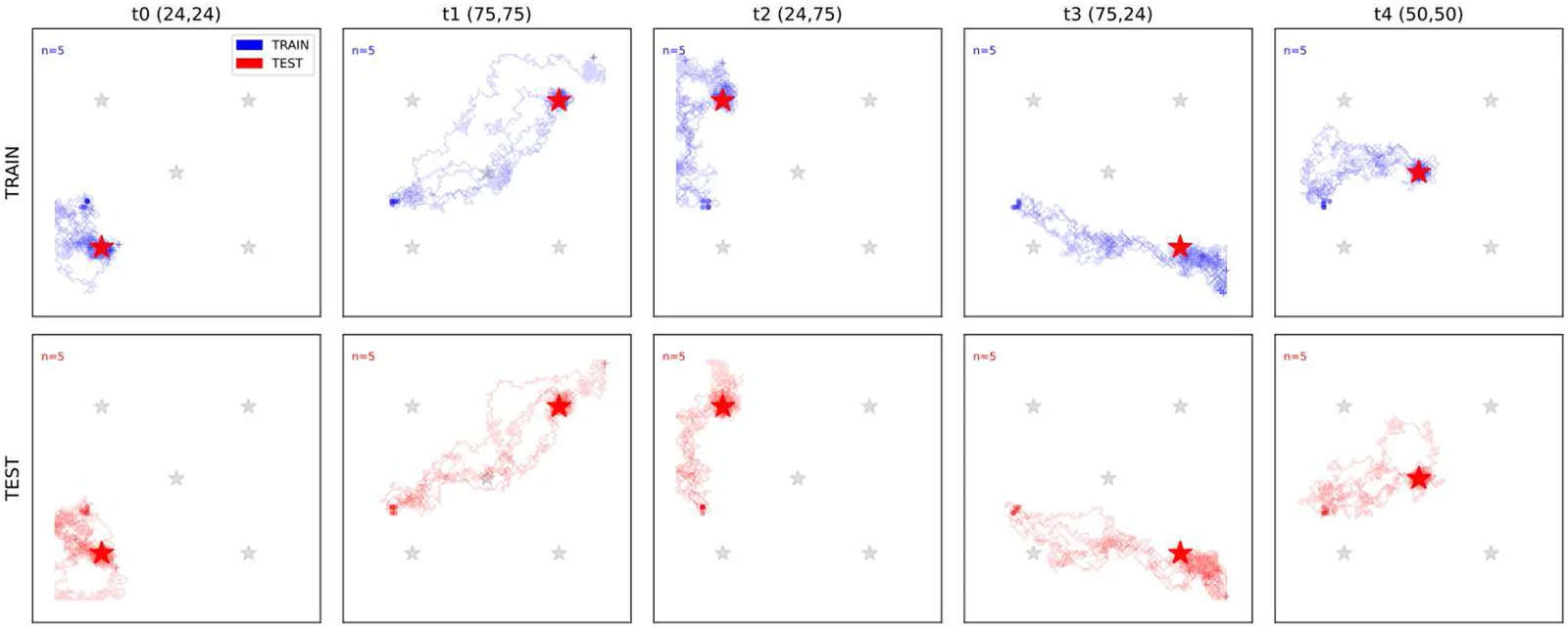
Representative late-learning trajectories during target-context evidence-gated plasticity (EGP). Example trajectories from a representative late-learning episode are shown separately for TRAIN and TEST phases. Columns correspond to the five target locations: t0 (24,24), t1 (75,75), t2 (24,75), t3 (75,24), and t4 (50,50). Blue trajectories show TRAIN-phase behavior and red trajectories show TEST-phase behavior. The active target is indicated by a large red star, whereas inactive target locations are shown as gray stars. Each panel contains five consecutive target blocks from the representative episode. The starting location is positioned in the left half of the environment, between the lower-left and upper-left targets, requiring the agent to distinguish the currently active goal from nearby competing targets. Across all targets, trajectories are directed toward the active target during both TRAIN and TEST phases, demonstrating stable target-selective navigation. Notably, late TRAIN-phase trajectories are already highly goal-directed despite ongoing proposal accumulation, indicating that behaviorally relevant navigation policies had largely been consolidated into the durable synaptic weight matrix by this stage of learning. TEST-phase trajectories exhibit similar target selectivity, consistent with successful evaluation and consolidation of accumulated synaptic proposals. Compared with the multi-target STDP/RL trajectories shown in Fig. 3, attraction toward inactive targets is substantially reduced. These examples provide a qualitative illustration of the improved target selectivity quantified by the dwell-time confusion analysis in Fig. 10.

To determine whether improved reward translated into improved navigation behavior, we examined representative late-learning trajectories from the target-context EGP model (Fig. 9). Across all five targets, both TRAIN and TEST trajectories were directed toward the currently active goal, with similar path structure observed between phases. Unlike the multi-target STDP/RL simulations, which frequently exhibited attraction toward previously learned targets, target-context EGP produced trajectories that remained focused on the active target and showed relatively little deviation toward inactive goal locations. Notably, the representative trajectories begin from a location in the left half of the environment, between the lower-left and upper-left targets. Despite starting near these competing goal locations, the agent reliably navigated toward the currently active target, including targets located on the opposite side of the environment. This effect was evident for both corner targets and the central target, although the central target continued to exhibit somewhat lower reward than the corner targets. One possible explanation for the reduced performance of the central target is that the center of the environment is traversed more frequently during exploration and during navigation to other targets. As a result, neural representations associated with the central target may overlap more extensively with representations used under other target contexts, increasing the potential for interference. Although the present study did not directly test this hypothesis, the consistently lower performance of the central target across analyses is consistent with greater representational overlap at this location.

Importantly, the similarity between late TRAIN and TEST trajectories does not indicate a lack of learning. Rather, by this stage of training, the EGP consolidation process had already embedded behaviorally beneficial weight changes into the durable synaptic matrix. Consequently, the network entered each TRAIN phase with an already effective navigation policy, resulting in high-quality trajectories and reward in both TRAIN and TEST conditions. The TEST phase therefore served primarily as a validation of previously consolidated synaptic modifications rather than the sole source of improved performance. The ability of the agent to reach the correct target even when starting near alternative goal locations further suggests that navigation behavior was guided by target-specific representations rather than simple attraction to the nearest learned target. Thus, by the late-learning stage shown here, reward improvements had generalized to the evaluation phase and were supported by stable synaptic changes rather than transient within-phase adaptation.

The spiking activity underlying these trajectories remained sparse throughout the network. Spatial representation populations exhibited low average firing rates (~1-2 Hz in EV, EVPlace, EGrid, and EPlace), while the association populations EAN, EAE, EAW, and EAS fired at only ~0.18 Hz on average. Motor-output populations likewise remained sparsely active, firing at approximately 0.43-0.47 Hz. In contrast, inhibitory populations maintained higher rates (typically 5-19 Hz), helping regulate overall network excitability. Thus, successful navigation emerged from coordinated activity across the circuit despite very low firing rates in the association and motor populations, demonstrating that sparse spiking activity was sufficient to support target-selective behavior and evidence-gated learning.

**Fig. 10.**
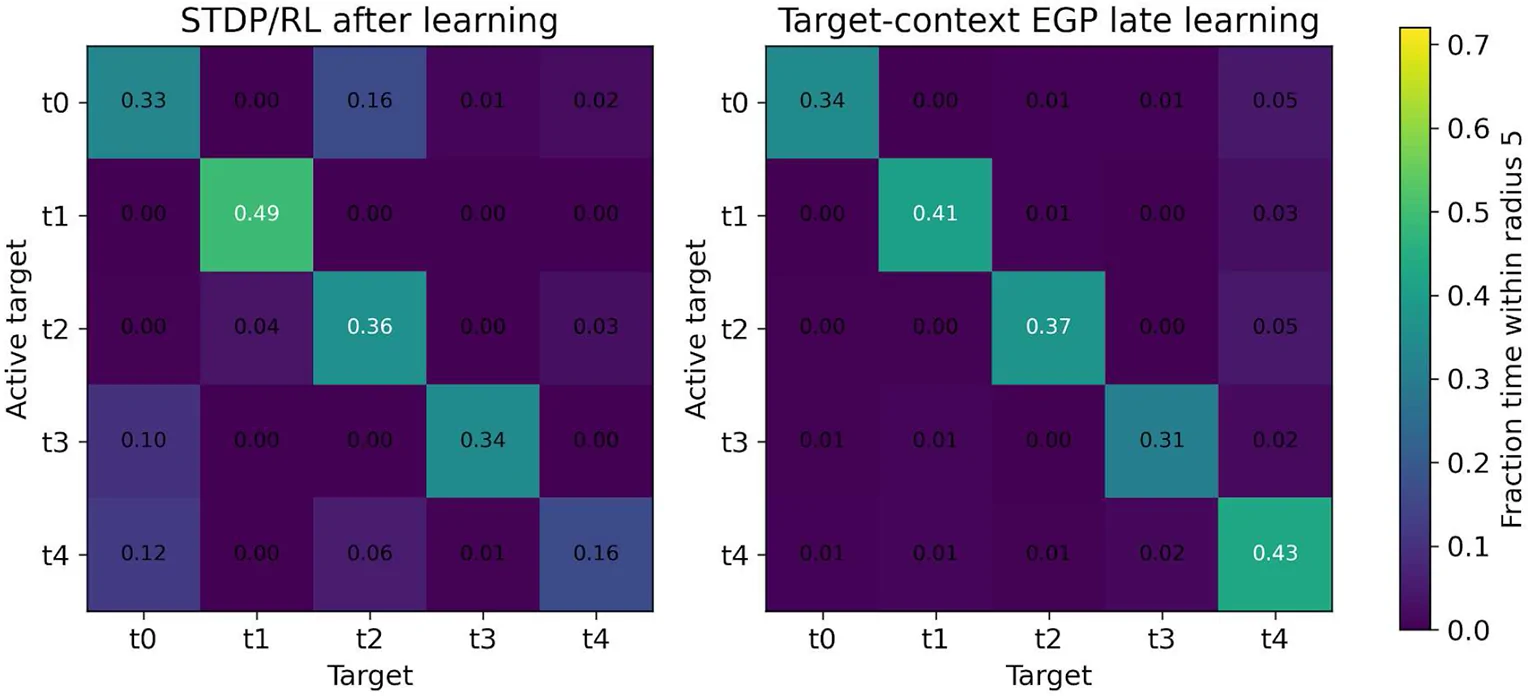
Target-context evidence-gated plasticity (EGP) reduces target confusion and improves navigation selectivity. Dwell-time confusion matrices quantify the fraction of each episode spent within a radius of 5 pixels from each target location. Rows indicate the active (intended) target, and columns indicate the target location occupied by the agent. Diagonal entries therefore represent correct target-specific occupancy, whereas off-diagonal entries represent attraction to incorrect targets. **A)** Multi-target STDP/RL after learning. Although the agent frequently occupied the intended target location, substantial off-diagonal occupancy remained, indicating interference between learned target policies and attraction toward competing goals. **B)** Target-context EGP during late learning. The confusion matrix exhibits stronger diagonal structure and reduced off-diagonal occupancy, indicating improved target selectivity and reduced interference between navigation contexts. Quantitatively, mean correct-target dwell increased from 0.337 under STDP/RL to 0.373 under target-context EGP, while the weakest-target diagonal dwell increased from 0.163 to 0.310. The largest effect was a reduction in wrong-target attraction: maximum off-diagonal dwell decreased from 0.163 to 0.049, and the ratio of mean diagonal to mean off-diagonal dwell increased from 11.96 to 31.46. These results demonstrate that target-context EGP improves multi-goal navigation primarily by reducing interference between learned target representations and increasing behavioral selectivity for the currently active goal.

Finally, we examined how the differences observed in the EGP experiments manifested behaviorally by comparing dwell-time confusion matrices from representative multi-target STDP/RL and target-context EGP simulations (Fig. 10). These analyses provide a detailed view of target selectivity and wrong-target attraction within individual network realizations. The EGP confusion matrix was computed from late-learning evaluation periods in which TRAIN and TEST trajectories had largely converged (Fig. 9), indicating that the observed target selectivity reflected consolidated navigation policies rather than transient within-phase adaptation. Under STDP/RL, the agent spent substantial time near incorrect targets, producing several prominent off-diagonal entries in the confusion matrix. For example, trajectories associated with target t0 frequently visited the vicinity of t2, while trajectories associated with t3 and t4 showed substantial dwell near t0. In addition, target t4 exhibited relatively poor target selectivity, with comparable occupancy near multiple target locations. These off-diagonal occupancies are consistent with partial entanglement of multiple target policies within the shared synaptic substrate.

In contrast, target-context EGP produced a substantially more diagonal confusion matrix, indicating more selective occupancy near the intended target and reduced attraction to incorrect targets. Qualitatively, the trajectories shown in Fig. 9 are consistent with this result, as the agent typically approached and remained near the active target rather than becoming trapped by alternative goal locations. The strongest improvements were observed for targets that exhibited substantial interference under STDP/RL. In particular, the central target (t4) increased from 0.16 to 0.43 correct-target dwell, while off-target occupancy was reduced across nearly all target pairs. Most off-diagonal entries under target-context EGP were close to zero, with the remaining residual confusion largely confined to weak interactions involving the central target.

Quantitatively, mean correct-target dwell increased from 0.337 under STDP/RL to 0.373 under target-context EGP, while the weakest-target correct dwell increased from 0.163 to 0.310. The largest effect was a reduction in off-target attraction: maximum off-diagonal dwell decreased from 0.163 to 0.049, and the ratio of mean diagonal to mean off-diagonal dwell increased from 11.96 to 31.46. Thus, target-context EGP not only improved average target occupancy, but also substantially increased the selectivity of the learned navigation policies. Although some target-dependent variability remained, particularly between corner and central targets, the resulting behavior was considerably more target-specific than that produced by multi-target STDP/RL. Together, these results indicate that target-context EGP mitigates multi-target interference by preserving target-specific credit assignment during synaptic consolidation, thereby enabling more stable and selective navigation policies.

The dwell-time confusion analysis was performed on the long-duration target-context EGP simulation because dwell-based measures require sufficient time for agents to reach and remain near target locations. In the shorter multi-seed EGP experiments, target blocks lasted only 7.5 s, often providing limited time for substantial target occupancy to develop. Consequently, reward-based measures were more sensitive indicators of learning performance in the short-duration experiments, whereas the longer simulation was better suited for characterizing target selectivity and wrong-target attraction through dwell-time analysis.

## Discussion

In this study, we developed a biologically grounded spiking neural network model of spatial navigation and examined how different reinforcement learning mechanisms support single- and multi-goal behavior. Standard reward-modulated STDP/RL successfully learned and retained a single-target navigation policy, demonstrating that the network could store goal-directed behavior in its synaptic weights (Fig. 2). However, extending the same mechanism to multiple targets produced substantial interference (Fig. 3). Although the agent acquired target-directed behavior during training, performance became uneven across targets and deteriorated when plasticity was reduced, with trajectories and dwell-time analyses revealing attraction toward incorrect target locations (Fig. 4,5).

To address this limitation, we introduced evidence-gated plasticity (EGP), a multi-timescale learning rule that separates rapid generation of candidate synaptic modifications from slower reward-based consolidation (Fig. 1). Global EGP improved learning relative to immediate online plasticity, while target-context EGP produced the strongest performance (Fig. 6). Across connectivity seeds, target-context EGP increased late-stage reward, improved weakest-target performance, and reduced wrong-target attraction (Fig. 6-10). Notably, improvements in both late-stage reward and weakest-target performance were observed in all 10 connectivity seeds, indicating that the benefits of context-specific proposal accumulation were highly consistent across network realizations. Because both EGP variants employed need-based weighting, these improvements are attributable primarily to context-specific proposal accumulation, reward evaluation, and consolidation. Together, the results indicate that the primary limitation of multi-target STDP/RL was interference arising from shared credit assignment across navigation goals.

The objective of this study was not to benchmark against a broad range of reinforcement-learning or continual-learning algorithms, but rather to isolate the contribution of evidence-gated consolidation and context-specific credit assignment within a common biologically inspired spiking architecture. Consequently, the principal comparisons focused on standard STDP/RL, global EGP, and target-context EGP. This progression allowed the effects of delayed consolidation and context-specific proposal accumulation to be evaluated while holding network structure, neural representations, and task conditions constant.

The main conclusion is that biologically inspired spiking navigation circuits can learn goal-directed behavior using local plasticity rules, but robust multi-goal learning benefits from mechanisms that preserve context-specific credit during consolidation. Standard STDP/RL provides rapid adaptation but applies updates immediately, allowing synaptic changes generated by different goals to interfere. EGP separates fast proposal generation from slower consolidation, enabling the network to evaluate whether accumulated changes improve behavior before they are committed. Target-context EGP further maintains separate proposal stores for different goals, reducing interference and improving behavioral selectivity.

This organization is consistent with the view that biological learning operates over multiple timescales[32,33]. Rapid STDP-like processes can modify synaptic efficacy during behavior, enabling adaptation to local contingencies, while slower mechanisms determine which changes are retained. Neuromodulatory gating, replay, synaptic tagging, and systems-level consolidation have all been proposed as contributors to memory stabilization[36,44-48]. EGP abstracts this principle into a computational framework in which local activity generates candidate modifications and longer-timescale reward evidence determines whether they are consolidated. The target-context component is similarly motivated by evidence that hippocampal, entorhinal, prefrontal, and striatal circuits encode contextual information that influences memory retrieval, action selection, and plasticity[4,49-52]. In the present model, target identity provides a simplified context signal that allows a shared spiking network to maintain distinct credit traces for different goals while retaining a common long-term synaptic substrate.

An additional biological interpretation of EGP is through the framework of synaptic tagging and capture (STC) and related behavioral tagging mechanisms[42,53-55]. In STC, transient synaptic activity establishes local tags that mark recently active synapses, while subsequent plasticity-related signals determine which tagged synapses undergo long-lasting stabilization. Viewed through this lens, the TRAIN phase of EGP resembles synaptic tagging, in which candidate synaptic modifications are generated and stored as transient proposals. The TEST phase provides a form of behavioral evaluation analogous to the availability of plasticity-related signals, determining whether the tagged modifications are associated with improved performance. Consolidation of proposals into the durable synaptic matrix then parallels the stabilization of tagged synapses through late-phase potentiation and memory consolidation[46]. Although EGP is not intended as a mechanistic model of STC, this correspondence suggests that synaptic tagging frameworks may provide a useful biological interpretation of how local plasticity and delayed behavioral outcomes can be linked during continual learning.

Evidence-gated signals are also biologically plausible at a functional level. Dopaminergic reward prediction error signals, acetylcholine-dependent state and attention signals, noradrenergic arousal signals, and hippocampal replay events could all influence whether recent synaptic modifications are strengthened, weakened, or stabilized[56-59]. The TEST phase in EGP is an algorithmic abstraction of this broader idea: candidate changes are not consolidated solely because they occurred during reward-modulated activity, but because they improve subsequent performance. Biological consolidation may occur during quiet wakefulness, replay, or sleep, when recent experiences are reactivated and evaluated in relation to reward, novelty, or task relevance[36,37]. Thus, EGP provides a bridge between online synaptic eligibility mechanisms and slower consolidation processes that may operate outside the immediate behavioral episode.

An important feature of the target-context EGP experiments is that learning occurred under a continual-learning protocol rather than a fixed start-to-goal mapping. Across 2000 sequential episodes, starting locations were randomized while synaptic weights persisted across episodes. The network therefore had to learn target-selective navigation policies that generalized across many initial conditions rather than memorizing specific trajectories. Within each episode, target contexts were repeatedly alternated during TRAIN and TEST phases, requiring the system to maintain and update multiple navigation policies over extended periods of learning. The resulting improvements in reward, trajectory quality, and confusion-matrix selectivity therefore reflect the acquisition of stable target-dependent behaviors under changing behavioral conditions. This setting is particularly relevant biologically, as animals routinely encounter familiar goals from novel locations and must integrate new experiences without catastrophically overwriting previously acquired memories. The observed reduction in target interference suggests that context-dependent consolidation may provide an effective mechanism for balancing memory stability and adaptability in neural systems[60].

The results also clarify the role of reward shaping in navigation learning. Stepwise rewards based on movement toward or away from the target provided a useful training signal and generally correlated with target-directed behavior. However, reward and target hits were not identical measures of performance. An agent could accumulate positive reward while following inefficient trajectories, or achieve repeated target hits while receiving lower average reward due to oscillatory or circuitous paths. This distinction likely contributes to the residual differences observed across targets, including the lower asymptotic reward achieved by the central target despite substantial improvements under target-context EGP.

Several limitations remain. First, the current model employs simplified spatial and target-related populations rather than fully detailed entorhinal-hippocampal circuitry[61,62]. Although the representations are biologically inspired, mechanisms such as recurrent hippocampal dynamics, theta-phase organization, replay, and neuromodulator-specific effects are not explicitly modeled[63]. Second, target-context EGP assumes that contextual information is available for gating plasticity. While biologically plausible, future work should investigate whether similar context signals can emerge from neural activity rather than being specified directly[64-66]. Third, target-context EGP reduces but does not eliminate interference, suggesting that additional representational factorization, more structured association populations, or adaptive upstream plasticity may further improve policy separation. In addition, the dwell-time confusion analyses were examined in greatest detail for representative simulations rather than across all network seeds. Although these analyses provide mechanistic insight into how target-context EGP improves target selectivity, future work should quantify confusion-matrix statistics across larger ensembles of network realizations. Finally, the simulations remain computationally expensive, limiting the scale of parameter sweeps and large multi-seed studies.

A related consideration concerns the interpretation of the STDP/RL and EGP comparisons. The multi-target STDP/RL and EGP experiments were not designed as fully matched algorithmic benchmarks. EGP introduces alternating TRAIN and TEST phases, proposal accumulation, and context-dependent consolidation, resulting in differences in learning dynamics and target scheduling relative to the STDP/RL experiments. Consequently, comparisons between STDP/RL and EGP should be interpreted as contrasts between alternative learning frameworks rather than strictly controlled ablations. The most direct comparison in the present study is therefore between global and target-context EGP, which share the same network architecture, reward structure, target schedule, and consolidation framework. Notably, the global and target-context EGP experiments used identical target schedules, suggesting that the performance differences observed between those conditions arise primarily from the proposal accumulation and consolidation mechanisms rather than target scheduling alone. Future work should examine matched training protocols to more precisely isolate the contributions of evidence-gated consolidation relative to standard online STDP/RL.

Future work should extend this framework to moving targets and changing goal contingencies, investigate neural mechanisms capable of generating context representations endogenously, and incorporate more biologically detailed consolidation processes such as replay-driven or sleep-like evaluation of candidate synaptic modifications[34]. More broadly, the principles underlying EGP, including rapid local eligibility, slower evidence-based consolidation, and context-specific credit assignment, may prove useful beyond spatial navigation, including continual learning in spiking networks, neuromorphic systems, and biologically grounded reinforcement learning architectures[67-75].

Overall, this work demonstrates that mammalian-inspired spatial representations and local plasticity rules are sufficient for learning goal-directed navigation, but that multi-goal learning exposes interference that standard online STDP/RL does not fully resolve. Evidence-gated plasticity stabilizes learning by separating proposal generation from consolidation, and target-context EGP further improves performance by preserving context-specific credit assignment. These findings support the broader hypothesis that multi-timescale, context-gated plasticity is an important component of continual learning in biologically realistic spiking neural networks.

## Supplementary Material

Supplement 1

## Figures and Tables

**Table 1.** Event-based integrate and fire neuron model parameters used in the spiking neuronal network.

Population class	Populations	RMP (mV)	VTH (mV)	Vblock (mV)	Refrac. (ms)	tauAHP (ms)	ahpwt	tauRR (ms)	RRW
Grid/target excitatory (non-adapting)	EGrid, EVGrid	−65	−40	−25	25	400	0	8	0.75
Place/target-place excitatory	EPlace, EVPlace	−65	−40	−25	25	400	1	8	0.75
Association excitatory	EAN, EAS, EAE, EAW	−65	−35	−25	25	400	5	8	0.5
Motor excitatory	EMN, EMS, EME, EMW	−65	−40	−25	100	400	5	100	0.5
Fast-spiking inhibitory	IV, IVPlace, IGrid, IPlace, IA, IM	−65	−40	−10	2.5	50	0.5	1.5	0.25
Low-threshold inhibitory	IVL, IVPlaceL, IGridL, IPlaceL, IAL, IML	−65	−40	−10	2.5	50	0.5	1.5	0.25

Connection delay parameters	
Parameter	Value
Somatic connection delay	Uniform (1.8, 2.2) ms
Dendritic connection delay	Uniform (2.0, 4.0) ms

**Abbreviations:** RMP, resting membrane potential; VTH, spike threshold; Vblock, depolarization block threshold; tauAHP, after-hyperpolarization decay constant; tauRR, relative refractory time constant; RRWght, relative refractory weight; tauAM, AMPA-like synaptic decay constant; tauNM, NMDA-like synaptic decay constant; tauGA, GABAA-like synaptic decay constant. EGrid and EV populations were configured as non-adapting excitatory neurons by setting ahpwt = 0 and increasing tauAM to the action-selection interval (50 ms), producing sustained location- and target-dependent activity during navigation. The neuron model is an event-driven point-neuron model and therefore does not contain explicit somatic or dendritic compartments. Nevertheless, synaptic inputs were assigned either short-delay (“somatic”) or long-delay (“dendritic”) pathways. These delay classes were used to approximate the longer integration and propagation times associated with synapses arising on proximal versus distal neuronal compartments in biological neurons. Thus, the somatic and dendritic labels refer to functional delay classes rather than anatomically explicit neuronal compartments.

**Table 2.** Network populations and configured input convergence.

Population	Neurons	Inputs/neuron	Source populations, convergence per postsynaptic neuron
EVGrid	384	29	IV (16); IVL (13)
IV	88	101	EVGrid (60); IV (22); IVL (19)
IVL	40	60	EVGrid (45); IV (12); IVL (3)
EVPlace	384	69	EVGrid (19); IVPlace (30); IVPlaceL (20)
IVPlace	88	102	EVPlace (60); IVPlace (22); IVPlaceL (20)
IVPlaceL	40	67	EVPlace (45); IVPlace (12); IVPlaceL (10)
EGrid	384	50	IGrid (30); IGridL (20)
IGrid	88	102	EGrid (60); IGrid (22); IGridL (20)
IGridL	40	67	EGrid (45); IGrid (12); IGridL (10)
EPlace	384	69	EGrid (19); IPlace (30); IPlaceL (20)
IPlace	88	102	EPlace (60); IPlace (22); IPlaceL (20)
IPlaceL	40	67	EPlace (45); IPlace (12); IPlaceL (10)
EAN	768	220	EVPlace (10); EPlace (10); IA (120); IAL (80)
EAS	768	220	EVPlace (10); EPlace (10); IA (120); IAL (80)
EAE	768	220	EVPlace (10); EPlace (10); IA (120); IAL (80)
EAW	768	220	EVPlace (10); EPlace (10); IA (120); IAL (80)
IA	704	416	EAN (60); EAS (60); EAW (60); EAE (60); IA (88); IAL (80); EMW (2); EME (2); EMS (2); EMN (2)
IAL	320	280	EAN (46); EAS (46); EAW (46); EAE (46); IA (48); IAL (40); EMW (2); EME (2); EMS (2); EMN (2)
EMN	192	88	EAN (10); IM (57); IML (21)
EMS	192	88	EAS (10); IM (57); IML (21)
EME	192	88	EAE (10); IM (57); IML (21)
EMW	192	88	EAW (10); IM (57); IML (21)
IM	176	356	EMW (61); EME (61); EMS (61); EMN (61); IM (80); IML (32)
IML	80	339	EMW (72); EME (72); EMS (72); EMN (72); IM (44); IML (7)

**Note:** Only populations with nonzero cell counts are shown. Convergence values indicate the intended number of presynaptic inputs from each source population onto each postsynaptic neuron.

**Table 3.** Plastic synapses and learning parameters.

Parameter	Value
Plastic projection(s)	EAN→EMN, EAS→EMS, EAE→EME, EAW→EMW
Synapse type	AMPA-like
Learning rule	Reward-modulated STDP (STDP/RL)
Eligibility duration	50 ms
STDP pre/post interval	100 ms
RL *ΔW* factor	0.045
Minimum weight	0
Maximum weight	12
Evidence-gated consolidation	Global or target-context EGP

**Table 4.** Summary of simulation protocols and computational run-times. Statistical analyses and figures were often based on selected subsets of episodes (e.g., early learning, late learning, and post-learning periods) rather than the full number of simulation episodes.

Simulation set	Episodes	Simulated time (days)	Runtime (days)
Single-target STDP/RL (learning + post-learning)	1600	3.47	1.09
Multi-target STDP/RL (learning + post-learning)	8000	17.36	5.46
Global EGP (10 seeds)	8000	34.72	10.93
Target-context EGP (10 seeds)	8000	34.72	10.93
Long-duration target-context EGP	2000	34.72	10.93
**Total**	**27,600**	**125.0**	**39.34**

## Data Availability

All data and source code supporting the findings of this study will be made available on github.com in a public open-access repository upon manuscript publication.
