# Supplementary material for: Context-Aware Evidence-Gated Plasticity for Multi-Goal Learning in Spiking Neural Networks
Source: Res Sq. 2026 Aug 4:rs.3.rs-10157488. Preprint. [Version 1] doi: 10.21203/rs.3.rs-10157488/v1 (PMC13484804; doi:10.21203/rs.3.rs-10157488/v1)
Supplement: Supplementary file 1 [file NIHPPrs10157488v1-supplement-1.pdf]

Supplementary Material

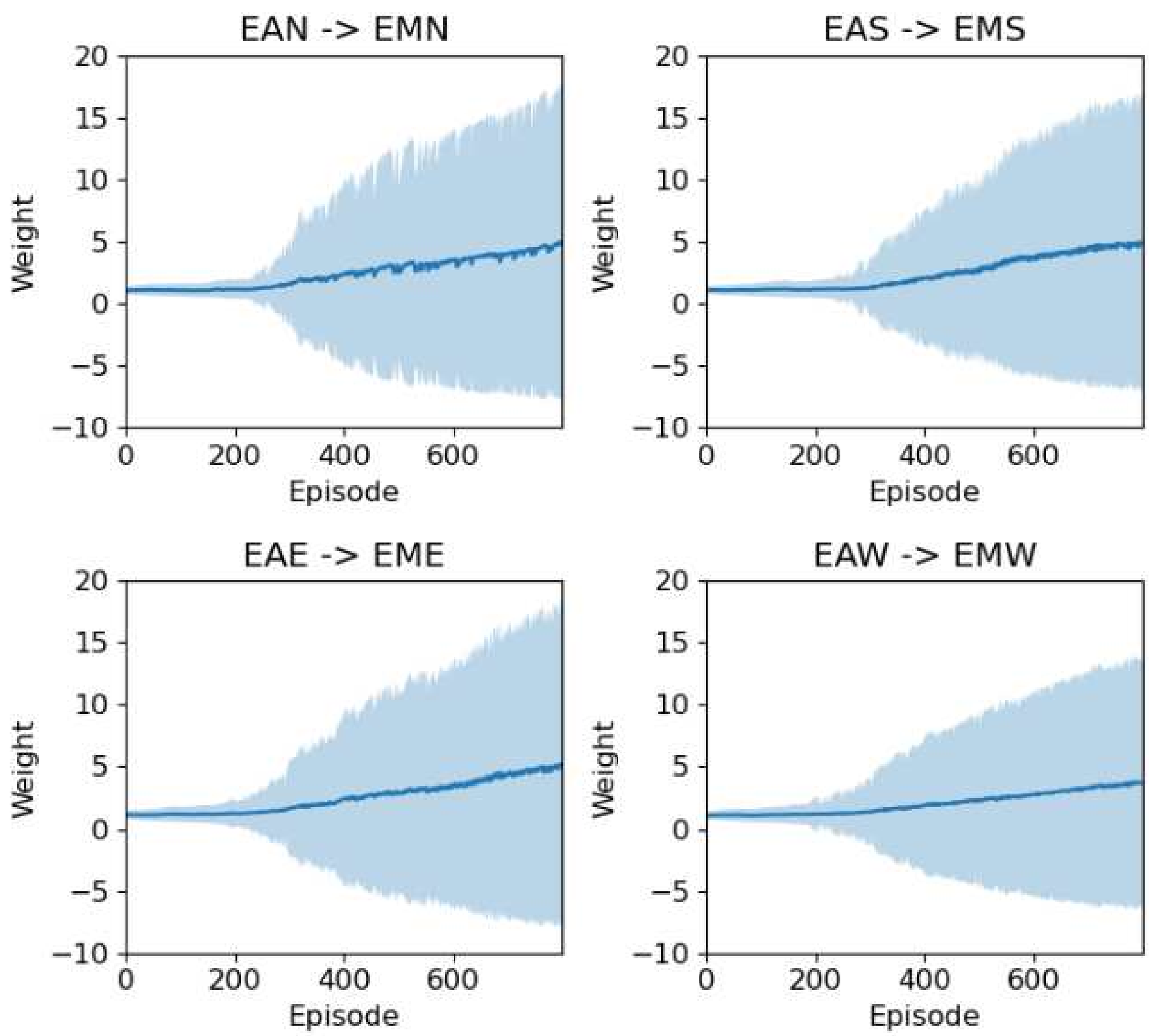

Supplementary Figure S1. Weights increase during STDP/RL learning of a single target, supporting post-learning performance. Solid line is average synaptic weight, +/- standard deviation (n=7680 synapses); Note that individual synaptic weights are bounded at or above zero; the shaded band extends below zero only because the standard deviation exceeds the mean at some time points, not because any weight becomes negative..
